# Comparison of Biochemical Constituents and Contents in Floral Nectar of *Castanea* spp.

**DOI:** 10.3390/molecules25184225

**Published:** 2020-09-15

**Authors:** Young Ki Kim, Sujin Lee, Jeong Ho Song, Mahn Jo Kim, Ural Yunusbaev, Myeong-Lyeol Lee, Mun Seop Kim, Hyung Wook Kwon

**Affiliations:** 1 Division of Special Forest Product, National Institute of Forest Science, 39 Onjeong-ro, Suwon 16631, Korea; treeace@korea.kr (Y.K.K.); sjh8312@korea.kr (J.H.S.); otttr@korea.kr (M.J.K.); 2 Department of Life Sciences & Convergence Research Center for Insect Vectors, Incheon National University, 199 Academy-ro, Incheon 22012, Korea; sujin0316@inu.ac.kr (S.L.); uralub@gmail.com (U.Y.); mllee6@inu.ac.kr (M.-L.L.)

**Keywords:** *Castanea*, nectar secretion, sugar content, amino acid contents, volatile organic compounds

## Abstract

Pollination is essential for efficient reproduction in pollinator-dependent crops that rely on the attraction of pollinators to flowers. Especially, floral nectar is considered to be an important factor attracting pollinator like honey bees, but differences among major chestnut species (*Castanea crenata*, *C. mollissima*, *C. dentata*, and *C. sativa*) are still little explored. This study aims to evaluate the value of honey source by analyzing floral nectar characteristics and comparing the composition of volatile organic compounds (VOCs) that mediate plant-pollinator interaction. In this study, we analyzed nectar samples obtained from male flowers using HPLC and HS-SPME/GC–MS. The five chestnuts showed significant differences between the volume of secreted nectar, free sugar composition, amino acid content and VOCs composition. Furthermore, *C. crenata* (Japanese cultivar ‘Ungi’) was revealed to emit the highest total amounts of VOCs and high levels of benzenoid compounds that are generally associated with flower-visiting insects. The sugar content per catkin, which is used to determine the honey yield, was the highest in *C. crenata*, suggesting that *C. crenata* ‘Ungi’ can be highly valued as a honey tree. Therefore, a better understanding of the relationship between pollinator and nectar characteristics of *C. crenara* could contribute to a prospective honey plant.

## 1. Introduction

Pollination is essential for efficient reproduction in pollinator-dependent crops that rely on the attraction of pollinators to flowers [1]. There are main factors that influence pollinators’ visits. The first is the morphological and anatomical structure of flowers such as size, color, flower organs, and the second is the volume and composition of the nectar and pollen produced by the flower [2,3]. In particular, pollinators such honey bees are directly provided with the primary source of energy through nectar and prefer better nutritive values, the qualitative and quantitative characteristics in the nectar are the most important for attracting pollinators as reviewed in [4,5,6]. The secretion of nectar takes place in a specially developed tissue called nectary. It is known that the mechanism of nectar secretion begins with photosynthesis and originates in phloem sap [7]. The sugar accumulated in parenchyma cells existing near the nectary causes the osmotic phenomenon and then is hydrolyzed. Therefore, nectar is composed of about 80% water and contains amino acids, organic acids, vitamins, and minerals [8].

**Figure 1:**
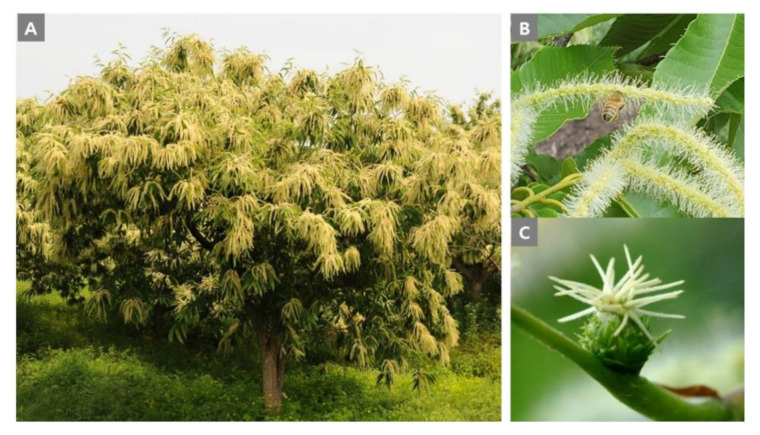
Picture of a chestnut tree including shape of tree (**A**), male and female flowers (**B**,**C**). The secretion of nectar is mainly from male flowers [11], and it can be found that male flowers are much more abundant than female flowers.

*Castanea* (Chestnut), belonging to the family Fagaceae, is distributed in over ten species in temperate climate regions, including Asia, Europe and North America. Among chestnut trees, *C. crenata, C. dentata, C. sativa,* and *C. mollissima* are important species consumed as a crop. In Korea, a chestnut pest (*Dryocosmus kuriphilus*) infection occurred in 1958, putting chestnut trees on the verge of extinction. To overcome the decline of the chestnut tree population, various chestnut tree species were introduced in 1965 from Japan, China, North America, and Europe to improve insect-resistance and fruit production. Currently, chestnut trees are distributed over about 77,440 ha and account for 21.3% of major non-timber products in Korea [9,10]. Chestnut trees are valuable not only as a food, but also as a honey source plant, being already classified as major honey plants in many countries [11]. The inflorescence is composed either of male or female flowers, or staminate and pistillate flowers occur together. Only the male flowers produce nectar that is secreted on the surface of the disc located at the base of filaments in staminate flowers (Figure 1), from which is possible to produce 27.2 kg of honey per 100 trees [11]. Chestnut honey was also identified to contain functional and specific substances: The content of kynurenic acid which shows anti-inflammatory and anti-oxidant effects was high in *C. sativa* honey [12]. Additionally, 1-phenylethanol and 2-aminoacetophenone are considered marker compounds of unifloral chestnut honey [13,14].

Volatile organic compounds (VOCs) that mediate plant-pollinator interaction has been evolved by pollinator behavior for reproductive success [15], and the VOCs of the chestnut flower have been reported in several studies to date [16,17,18]. Even though floral nectar is considered to be an important factor attracting pollinators like honeybees, little is known about pollinator attractant factors in major chestnut species yet. Hence, this study aims to evaluate the value of honey source by analyzing floral nectar characteristics, as well as comparing the composition of VOCs. We collected floral nectar in *Castanea* spp., such as *C. crenata* (Korean cultivar ‘Mipung’, CCK; Japanese cultivar ‘Ungi’, CCJ), *C. dentata* (America sweet chestnut, CDA), *C. mollissima* (Chinese chestnut, CMC), and *C. sativa* (European chestnut, CSE), from extension forest in National Institute of Forest Science (Suwon, Republic of Korea) that were planted in 1995. Our work could contribute to a comprehensive understanding about breeding for high-quality and increasing honey production within chestnut trees.

## 2. Results

### 2.1. Analysis of Nectar Volume and Free Sugar Content

The results of examining nectar volume, free sugar content, sugar composition, and sugar content per catkin in floral nectar of the *Castanea* spp. are shown in Table 1. There were differences in flowering date from June 19 to 21 for CCK, CCJ and CDA, June 25 to 27 for CMC, and June 27 to 29 for CSE. The nectar volume per catkin (μL/catkin) was highest in CCJ with 42.7 ± 10.8 μL, followed by CCK (20.9 ± 7.6), CCM (11.0 ± 3.0), CSE (10.7 ± 3.4) and CDA (8.7 ± 2.4). Also, the Kruskal-Wallis H-test showed significant differences in the nectar volume per catkin among the individuals (*p* = 0.000). The nectar concentration ranged from 22.8 to 61.8 Brix, and the free sugar content ranged from 22.6 to 82.5 μg. Both characteristics were highest in CMC, indicating a significant difference between individuals (*p* = 0.000). The sucrose/hexose ratio ranged from 0.02 to 0.17, indicating the hexose dominant grade (CCK, CSE) or hexose-rich grade (CCJ, CDA, CCM). In addition, the sugar content per catkin, which is known to be relatively less affected by environmental factors, was found to be 0.31 to 1.35 mg/catkin, indicating a significant difference between individuals (*p* = 0.000).

### 2.2. Correlation between Nectar Characteristics and Meteorological Factors

As a result of analyzing the Spearman correlation between the properties of nectar and weather conditions, eight correlations were confirmed (Table 2). Temperature and relative humidity were not correlated with nectar volume per catkin. In contrast, temperature showed a strong correlation with nectar concentration (0.772) and free sugar content (0.716), and relative humidity showed a negative correlation with nectar concentration (−0.729) and free sugar content (−0.729). The nectar volume per catkin was correlated with nectar concentration (−0.631), free sugar content (−0.681), and nectar sugar content per catkin (0.556). Also, the nectar concentration was strongest correlated with free sugar content (0.907).

### 2.3. Analysis of Amino Acid Content

The results of amino acid content in the floral nectar of *Castanea* spp. are shown in Table 3. A total of 20 amino acids were detected in chestnut nectar, the highest in CMC (118.1 ± 27.6 mg/L), and the lowest in CCK (53.9 ± 12.6 mg/L). CCK, CCJ, and CDA had the highest proline (22.3~29.6%), while CMC and CSE had the highest asparagine (32.4~41.0%). Also, we have confirmed that it contains ten essential amino acids for honeybees. As a result of conducting the Kruskal-Wallis H test, significant differences were observed between the five amino acids asparagine, GABA, glutamine, proline and tryptophan (*p* < 0.05).

### 2.4. Floral Nectar VOCs Composition

**Figure 2:**
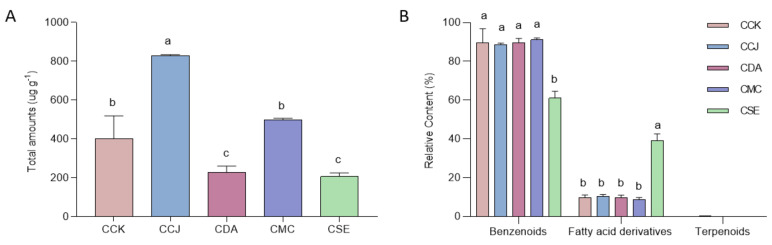
(**A**) Total amount (μg/g) of VOCs and (**B**) relative content (%) of VOCs classes in floral nectar of *Castanea* spp. The different letters on the bars indicate significant differences among *Castanea* spp. based on One-way ANOVA with Duncan’s multiple range tests (*p* < 0.05). Each value represents the mean ± SEM (*n* = 3). Abbreviations; CCK, *C. crenata* (Korean cultivar ‘Mipung’); CCJ, *C. crenata* (Japanese cultivar ‘Ungi’); CDA, *C. dentata* (American chestnut); CMC, *C. mollissima* (Chinese chestnut); CSE, *C. sativa* (European chestnut).

GC-MS analysis revealed 22 VOCs in the SPME extracts from chestnut nectar. Chemical analysis remarkably showed differences in the composition of several VOCs among *Castanea* spp. (Table 4). The average total amounts of VOCs significantly differed among the individuals (F_(4,7)_ = 28.793, *p* < 0.001; Figure 2A). The highest quantity of VOCs was emitted by CCJ with 829 ± 6 μg/g, followed by CMC (498 ± 9 μg/g), CCK (404 ± 116 μg/g), CDA (227 ± 33 μg/g) and CSE (207 ± 18 μg/g). Since floral VOCs can be divided into three major chemical classes (benzenoids, fatty acid derivatives, and terpenoids) based on their biosynthetic origin [19], the VOCs emitted from nectar were 12 benzenoids, nine fatty acid derivatives, and one terpenoid. Similar trends were observed for the quantities of benzenoids and fatty acid derivatives in CMC, CDA, CCK and CCJ, except terpenoids which only exist in CCJ, whereas CSE significantly differed compared to others (Figure 2B).

**Figure 3:**
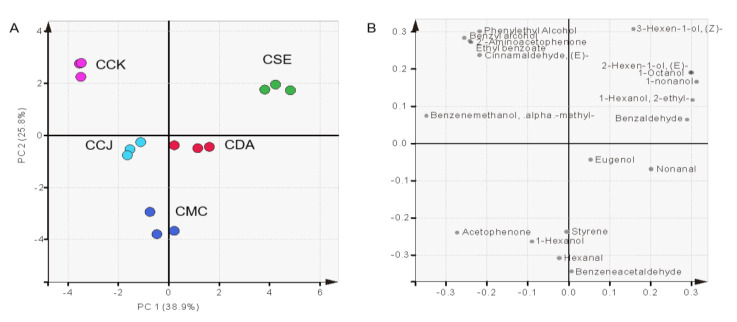
Score (**A**) and loading (**B**) plot of volatile compounds according to *Castanea* spp. Abbreviations; CCK, *C. crenata* (Korean cultivar ‘Mipung’); CCJ, *C. crenata* (Japanese cultivar ‘Ungi’); CDA, *C. dentata* (American chestnut); CMC, *C. mollissima* (Chinese chestnut); CSE, *C. sativa* (European chestnut).

Principal component analysis (PCA) was performed to find the differences in VOCs across chestnut species. The first principal component (PC 1) represented 38.9% and the second PC (PC 2) 25.8%, which explained 64.7% of the total variance. A scatter plot of the PCA scores (Figure 3A) showed the distribution of each *Castanea* spp., and the corresponding loading plot (Figure 3B) showed the VOCs. The differences of the volatile profiles have been shown clearly amid *Castanea* spp. The CSE and CCK were separated by PC1. The CSE had PC2 scores positive, attributed to the compounds distributed in that quadrant, especially fatty acid derivatives.

## 3. Discussion

In this study, we have analyzed the volume of secreted nectar, free sugar composition, amino acid content, and composition of VOCs in floral nectar obtained from *Castanea* spp. to evaluate the value of the honey source.

The nectar volume and nectar concentration affect the feeding behavior of pollinators [20,21,22]. In this study, significant differences in nectar volume (8.7~42.7 μL/catkin) and nectar concentration (22.8~61.8 Brix) were observed among the *Castanea* spp. (Table 1). Given that honey bees can collect nectar with a concentration of 15 to 60 Brix, the chestnut nectar is considered positive for honey bees [23]. According to Burquez and Corbet [24] that are differences in the nectar volume in the same species depending on the tree’s age, the location of flowers, the size of flowers, and environmental factors. Also, there was a difference in nectar volume according to soil type and moisture content [25]. In this study, the environmental variation was minimized by planting each species in the same location. It was determined that there was a difference in the nectar volume by chestnut species. A previous study was reported that there was a significant difference in the nectar volume and sugar concentration between Korea hawthorn (1.4 μL, 27.2 Brix) and Chinese hawthorn varieties (3.6~5.4 μL, 11.3~12.6 Brix) [26]. The result of investigating the nectar properties of four varieties of the *C. crenata* showed that nectar volume (6.7~54.3 μL) and nectar concentration (18.2~57.1 Brix) displayed significant difference among the varieties, showing the same trend as in this study.

Because the dietary sugar consumed by bees affects the amount of honey available to humans through digestive physiology, sugar analysis is required for study of honey plants [26,27]. In this study, the sugar content (22.6~82.5 μg) and sugar content per catkin (0.43~1.35 mg/catkin) of floral nectar showed a significant difference (Table 1, *p* = 0.000). A previous study suggested that the sugar content per flower calculated by using the nectar volume and free sugar content rather than the nectar volume affected by meteorological factors should be the main factor for investigation or individual selection of honey plants [28]. Free sugar content was highest in CMC, but sugar content per catkin considering the amount of nectar volume was highest in CCJ. As a result of analyzing the free sugar content of four chestnut varieties [27], the free sugar content (16.4 to 69.3 μg) and the sugar content per catkin (0.48 to 1.40 mg/catkin) displayed significant differences, which showed the same results as our study. However, a previous study comparing nectar characteristics between Korean and Chinese Hawthorn reported that the nectar volume and the free sugar content significantly differ, but the nectar sugar contents per flower of Korean hawthorn (69.2 μg) and Chinese hawthorn varieties (50.6~79.0 μg) did not significantly differ [26]. Therefore, it is judged that nectar characteristics such as the nectar volume, nectar concentration, and sugar content per catkin vary greatly depending on the species or varieties.

The sugar composition, such as the sucrose/hexose ratio (S/H ratio), affects the diet of pollinators [7,20]. According to the previous study [20], S/H ratio were classified into four grades; sucrose dominant (ratio > 1.0), sucrose rich (0.5–1.0), hexose rich (0.1–0.5), and hexose dominant (ratio < 0.1). In this study, the S/H ratio in the floral nectar of *Castanea* spp. ranged from 0.02~0.07 and showed hexose rich or hexose dominant grades (Table 1). Kim et al. [29] reported that the sucrose content in floral nectar of male and female *Evodia daniellii* Hmsl (Korean evodia tree) was 71.4% and 52.8%, respectively, and the S/H ratio was 2.5 and 1.2. Considering that the S/H ratio showed 0.6 in hawthorn and 0.2–0.7 in *Tilia insularis* Nakai (linden), it was found that chestnut nectar has a significantly lower S/H ratio [26,28]. In general, it is known that long-tongued pollinators such as honey bee prefer sucrose rich nectar, and short-tongued pollinators prefer hexose rich nectar [20]. Although there is no quantitative data, the number of visits by honey bees was relatively lower in chestnut than other honey plants because chestnut trees are pollinated by wind rather than insect-pollinator.

Generally, the nectar volume and nectar concentration are affected by temperature and relative humidity. Moderately elevated temperatures may increase nectar secretion, but strongly elevated temperatures reduce it [30,31]. For example, plants with an optimal temperature of 20–25 °C for nectar secretion have reduced nectar secretion at strongly higher temperatures. It means that the amount of nectar is regulated when it is out of the optimal environment for nectar secretion, although the optimum range of temperature for nectar secretion is known in only a few species [32]. There was no correlation between nectar volume and environmental factors in this study (Table 2). This means that the temperature during the flowering period is within the optimum range for the nectar secretion of chestnut trees. The nectar concentration was correlated with temperature (0.772) and relative humidity (−0.729). The low relative humidity evaporates moisture and concentrate nectar, while the high relative humidity tends to dilute the nectar [24,31]. As a result of examining the correlation between the nectar characteristics and the weather condition of the Korean evodia tree [29], It was reported that temperature correlated with nectar concentration (0.670) and the relative humidity (−0.802), showing a similar pattern to this study. In the correlation between nectar properties and weather factors of hawthorn [26], the nectar volume correlated with nectar concentration (−0.763) similar to this study. But, it was reported that there was a positive correlation between the nectar volume and the relative humidity (0.582), which was different from this study. The reason why there is no particular correlation pattern between the environmental factors and nectar characteristics in several honey plants is that the optimal environment for nectar secretion is different for each plant.

Although amino acids account for a relatively small proportion of nectar, they play an important role in determining the taste of nectar and attracting pollinators [22,33]. Essential amino acids for honey bees have been reported to be ingested from the food (nectar) because they cannot be synthesized in the honeybee’s body or converted from other amino acids [34]. In this study, ten essential amino acids have been detected (Table 3), suggesting that honeybees will prefer floral nectar of chestnut. According to the previous study, honey bees willingly give up sugars to acquire essential amino acids. In the case of phenylalanine, honey bees were willing to give up 84 units of sucrose for 1 unit of amino acid [35]. This means that bees are not absolutely affected by the sugar composition of nectar and are willing to visit chestnut flowers for other nutrients such as amino acids. Proline was found to be the highest amino acid in CCK, CCJ, and CDA, and asparagine was found to be the highest amino acid in CMC and CSE. Proline, non-essential amino acid, is a vital amino acid for the laying of queen bees, is necessary for the development of wing muscles of insects. It is also rapidly metabolized and results in the production of multiple nicotinamide adenine dinucleotide phosphate (reduced form) equivalents and high adenosine triphosphate (ATP). No other amino acid can be metabolized as rapidly as proline and release as much ATP without complete metabolism [34]. Because of this, proline is known to be a preferred ingredient of honeybees. Asparagine is known in the 73 Mediterranean plants as an amino acid that disgusts pollinators [35]. The hawthorn (15.7~17.1%) and the cherry tree (18.7~19.9%), which are known as honey plants, also showed a high content of asparagine, so it is judged that asparagine does not directly show aversion to bees [26,36]. Five amino acids such as asparagine, GABA, glutamine, phenylalanine, and proline showed differences between species (Table 3). Kim et al. [27] investigated the amino acids of floral nectar from four chestnut varieties (*C. crenata*) and reported that there were differences in 9 amino acids depending on the varieties. The reason for the difference in amino acid comparison is that the previous study performed statistical analysis using ANOVA test, whereas this study used the Kruskal-Wallis H-test. But, the critical point here is that the composition of nectar such as free sugars and amino acids varies depending on variety or species.

Flowering plants-pollinator relationships have been shaped by mutualistic associations [37]. Floral volatile emissions are a key phenotype that operate as advertisements to attract pollinators and mediate reproduction in angiosperms [38,39]. The pollinators must perceive VOCs that act as “honest floral signals”, advertising the presence of rewards, in order to gain an advantage from the high quality and quantity reward (e.g., nectar, pollen) [40]. The reward itself can emit the most obvious honest floral signals that play a vital role used by pollinators to identify and discriminate among flowers during foraging [41,42,43]. Honeybees have the ability to use the floral volatiles to distinguish subtle differences among flowers that depend on both intensity of the floral scent and the ratios of the concentrations of VOCs of a complex mixture [44].

The *Castanea* spp. differed not only in the total amounts of floral nectar VOCs, but also in the composition and quantity of several distinct compounds. Our result showed that the nectar of CCJ emitted the highest total amounts with 829 ± 6 μg/g. In a commercial strawberry field, the most abundant wild bee, *Osmia bicornis*, visited much more frequently strawberry flower varieties (Sonata) with the highest amounts of VOCs emitted [45]. Thus, our result may suggest that the most of the insects will be attracted by CCJ among others, yet this proposal should be supported by observing how the quantitative variation of VOCs affects ecological roles of pollinators.

We identified 22 of the VOCs detected on the nectar in the *Castanea* spp. (*n* = 3). The significant differences were observed between CSE and the others based on the relative content of VOCs. Benzenoids represented the most abundant class and included 12 individual compounds. Benzenoids are known to show a much more special relationship with pollinators than others, as well as particularly positive associations with Apidae [19]. Using bioassays and trapping experiments, it has been demonstrated that benzenoids may be greatly attractive to honey bees [46,47]. This chemical class was dominated by the presence of acetophenone, except for CSE, which is usually found mainly in the anthers presenting the pollen [48]. In previous studies, it was reported that the greater part of compounds emitted from chestnut flower were composed of acetophenone and 2-phenylethanol [17,18]. VOCs from anthers or pollen may function as either attractant for flower pollinators or defense against herbivores and/or pathogens [49]. In particular, Benzeneacetaldehyde, which accounts for about 21% in CMC, is known as an honest signal from bumblebee (*Bombus terrestris*), who increased preference for benzeneacetaldehyde over other VOCs after foraging on *Brassica rapa* [40]. Our work also proposes further research to reveal what VOCs are used by honey bees searching pollen or nectar as honest signals for *Castanea* spp., which independently reflects the status of each reward.

Fatty acid derivatives also represent a major chemical family involved in signaling molecules in intra- and inter-plant communication such as (*E*)-2-hexenal, (*Z*)-3-hexenol and methyl jasmonate, which are abundant in the plant kingdom and constitute another large class of plant volatiles [50]. This family was dominated by (*Z*)-3-hexen-1-ol in CSE, which is known to be typically emitted after mechanical damage and attract natural enemies of the herbivores, like wasps [51,52].

Different concentrations of distinct VOCs have been reported to influence the frequency of honey bee visitation to sunflowers [53] and oilseed rape [54] varieties, as well as to lead to different *O. bicornis* responses [45]. The relative quantity of certain compounds, creating subtle differences in floral scents, might be a driver for honeybees to recognize the distinctiveness among floral scents resulting in changes in the efficiency of foraging for pollen and nectar [55]. Knowing how pollinators perceive the subtle differences among flowers plays a significant part to understand the function of a floral scent as a signal. Therefore, additional olfactory experiments are needed to test how the honeybee’s behavioral response differs from its unique mixtures and total quantity of volatile emissions.

## 4. Materials and Methods

### 4.1. Plants Materials

The *Castanea* spp.-*C. crenata* (Korean cultivar ‘Mipung’, CCK; Japanese cultivar ‘Ungi’, CCJ), *Castanea dentata* (American sweet chestnut, CDA), *C. mollissima* (Chinese chestnut, CMC), and *C. sativa* (European chestnut, CSE)-were planted in experiment forest in National Institute of Forest Science (Suwon, Korea) in 1995. The average height of *Castanea* spp. was 4.5 m, and the diameter at breast height was 24 cm. The floral nectar of each chestnuts was collected at the peak of blossom from three individual trees in June 2019.

### 4.2. Collection of Floral Nectar and Investigation of Nectar Volumes

Three branches were selected from each individual in the same position. Nectar feeding was prevented by excluding honeybees and other insect pollinators using pollination bags. Nectar was gathered by centrifugation at 2000 rpm for 6 min. The nectar samples were then quantified using a 100 μL syringe (Hamilton Co., Reno, NV, USA). Sugar concentrations in nectar were measured using a GMK-703T portable saccharometer (Giwon Hitech, Seoul, Korea). Pure nectar obtained using a 0.45 μm membrane filter (#M9285, Millipore, Billerica, MA, USA) were stored at −70 °C in Eppendorf vials containing 80% ethanol (*v*/*v*%).

### 4.3. Analysis of Free Sugar Contents

Nectar samples filtered using a 0.45 μm syringe filter were analyzed using HPLC (Dionex ultimate 3000; Dionex, Sunnyvale, CA, USA). The mobile phase was deionized water at a flow rate of 0.5 mL/min and the oven temperature was 80 °C. Free sugars were identified using a refractive index detector (Ri-101, Shodex, Osaka, Japan) and an Aminex 87P column (300 × 7.8 mm, Bio-Rad, Hercules, CA, USA). Contents were calculated using an external standard method (linear regression equation, R^2^ > 0.999) with an integral meter and sucrose, glucose and fructose (#84097, sucrose; #49139, glucose; #47739, fructose, Sigma Aldrich, St. Louis, MI, USA) as standards.

### 4.4. Analysis of Amino acid Contents

The amino acids from nectar were analyzed using *o*-phthalaldehyde (OPA)-fluorenylmethyl chloroformate (FMOC) derivatization. The samples were mixed in borate buffer with OPA/mercaptopropionic acid (MPA) and FMOC, and filtered through a 0.45 μm syringe filter. Samples were then analyzed using an Agilent 1200 series HPLC instrument (Agilent Technologies, Santa Clara, CA, USA). The mobile phase comprised solutions A (10 mM Na_2_HPO_4_ and 10 mM Na_2_B_4_O_7_·10H_2_O, pH 8.2) and B (water: acetonitrile: methanol = 10:45:45, *v*/*v*%) and the gradient was set to 55:45 (*v*/*v*%) at 26–28 min from the initial 100:0, 0:100 from 28–30.5 min and 100:0 from 30.5 min. The C_18_ column (5 μm; Innopia, Seongnam-si, Korea) was used by setting the velocity of the 4.6 × 150 mm to 1.5 mL/min, the amount of injection to 1 μL and the column temperature to 40 °C. Ultraviolet rays were detected at 338 nm by connected UV detectors. The emission and excitation wavelengths measured using fluorescence were 450 and 340 nm for the OPA derivative and 305 and 266 nm for the FMOC derivative. The concentration of amino acid in the nectar was determined from calibration curves for the different amino acids (linear regression equation, R^2^ > 0.997). In order to compare the mean of the content ratio of 20 amino acids targeting floral nectar of chestnut, Kruskal-Wallis H test were carried out.

### 4.5. Headspace Solid-Phase Microextraction (HS-SPME) Sampling

A solid-phase microextraction (SPME) fiber (85 μm polyacrylate fiber, Supelco Inc., Bellefonte, PA, USA) was used to collect the nectar volatiles. The peak area of most of the target compounds was higher when they were extracted by 85 μm polyacrylate fiber than that of 65 µm PDMS/DVB fiber at equal extraction times, thus 85 μm polyacrylate fiber was selected as optimal. The 200 µL of nectar sample was diluted with 1 mL of deionized distilled water in a 20 mL headspace glass vial (Agilent Technologies) [56]. The vial was tightly capped with a 20 mm HS AL crimp cap (Agilent Technologies) and equilibrated for 30 min at 40 °C in a warmer. An SPME fiber was exposed and maintained above the sample surface for 30 min.

### 4.6. Volatile Organic Compounds (VOCs) Identification

The volatile profile analysis was performed on a GC-MS 7890B/5977B quadrupole, with electron ionization (Agilent Technologies). The separation was performed on Agilent HP-5MS analytical column (30 m × 0.25 mm, 0.25 μm) using 99.999% high purity helium as the carrier gas velocity in 36.262 cm sec^−1^. SPME fiber was injected into the GC injection port and the volatiles in fiber were desorbed onto the column at 250 °C for 3 min in splitless mode. The oven temperature was held at 40 °C for 6 min, increasing at a ratio of 6 °C min*−1* until 250 °C at 6 °C min*−1* (6 min hold). The mass spectrometer operated at 70 eV ionization energy. The ion source temperature was 280 °C. The identification of VOCs was performed by comparison of mass spectra with those in the mass spectra library (NIST/EPA/NIH 2017 Library) with the MSD Chemstation software (Version F.01.03.2357, Agilent Technologies). Linear retention indices (LRI) were calculated as a parameter for identifying compounds using a series of C8-C20 alkanes (#04070, Sigma Aldrich, St Louis, MO, USA), analyzed under the same chromatographic conditions. The semi-quantification of VOCs was calculated based on the corresponding calibration curves obtained by an external standard. A linearity ranges from 0.01, 0.1, 1 mg/mL of acetophenone (#42163, Sigma-Aldrich) was observed for the external standards (linear regression equation y = 46617x + 2E+06, R^2^ = 0.9971). Peak areas were normalized as percentage and used to determine the relative amounts of the VOCs. The data were expressed as mean ± standard error of the mean (SEM) of triplicate measurements. One-way analysis of variance (ANOVA) with Duncan’s multiple range tests to assess differences in VOCs among five different floral nectar of *Castanea* spp. using SPSS^®^ Statics 25 (IBM, Armonk, NY, USA). Principal component analysis (PCA) was employed to establish relationships between the *Castanea* spp. and their VOCs using the online software MetaboAnalyst 4.0 (http://www.metaboanalyst.ca). The data acquired from GC-MS was scaled with unit variance before all variables were subjected to the PCA. Figures were prepared in GraphPad^®^ Prism 8 (GraphPad Software, Inc., La Jolla, CA, USA).

## Figures and Tables

**Table 1:** Comparison to nectar volume, free sugar content, composition of sugar, and sugar content per catkin in floral nectar of *Castanea* spp.

Characteristics	CCK	CCJ	CDA	CMC	CSE	H Test *
Flowering date	June 19–21	June 19–21	June 19–21	June 25–27	June 27–29	
NVC (μL/catkin)	20.9 ± 7.6 ^b^	42.7 ± 10.8 ^a^	8.7 ± 2.4 ^c^	11.0 ± 3.0 ^b^	10.7 ± 3.4 ^b^	*p* = 0.000
NC (Brix)	22.8 ± 8.0 ^c^	34.2 ± 12.5 ^bc^	40.3 ± 6.2 ^b^	61.8 ± 5.9 ^a^	43.8 ± 14.6 ^bc^	*p* = 0.000
FSC (μg/μL)	22.6 ± 9.4 ^c^	40.1 ± 14.8 ^bc^	52.3 ± 14.6 ^b^	82.5 ± 16.8 ^a^	56.9 ± 15.2 ^bc^	*p* = 0.000
Sucrose (%)	3.7 ± 1.2 ^c^	7.9 ± 4.7 ^b^	13.4 ± 1.5 ^a^	15.9 ± 4.9 ^a^	3.2 ± 0.9 ^c^	*p* = 0.000
Glucose (%)	21.5 ± 1.4 ^bc^	20.5 ± 1.3 ^c^	33.7 ± 0.5 ^a^	22.6 ± 0.5 ^b^	32.7 ± 1.0 ^a^	*p* = 0.000
Fructose (%)	74.8 ± 1.9 ^a^	71.7 ± 3.5 ^b^	52.9 ± 1.3 ^d^	61.5 ± 5.4 ^c^	64.1 ± 1.7 ^c^	*p* = 0.000
S/H ratio	0.04 ± 0.02 ^c^	0.13 ± 0.10 ^b^	0.15 ± 0.01 ^a^	0.17 ± 0.06 ^a^	0.02 ± 0.00 ^c^	*p* = 0.000
SCPC ^1^ (mg/catkin)	0.45 ± 0.2 ^b^	1.35 ± 0.5 ^a^	0.43 ± 0.1 ^b^	0.93 ± 0.4 ^a^	0.31 ± 0.1 ^b^	*p* = 0.000

Data represent the mean ± SD (*n* = 9). * Kruskal-Wallis H test. The different letters within a row indicate significant differences among *Castanea* spp. based on Kruskal-Wallis H test with Dunn’s multiple comparison tests (*p* < 0.05). 1 multiply secreted nectar volume (μl/flower) and free sugar content per unit volume (μg/μL). Abbreviations; NVC, nectar volume per catkin (μL/catkin); NC, nectar concentration (Brix); FSC, free sugar content (μg/μL); S/H ratio, sucrose/hexose ratio; SCPC, sugar content per catkin (mg/catkin); CCK, *C. crenata* (Korean cultivar ‘Mipung’); CCJ, *C. crenata* (Japanese cultivar ‘Ungi’); CDA, *C. dentata* (American chestnut); CMC, *C. mollissima* (Chinese chestnut); CSE, *C. sativa* (European chestnut).

**Table 2:** Correlation coefficients by Spearman between meteorological factor and nectar characteristics.

Characteristics	NVC	NC	FSC	SCPC
Temperature	ns	0.772 **	0.716 **	ns
Relative humidity	ns	−0.729 **	−0.729 **	ns
NVC		−0.631 *	−0.681 **	0.556 *
NC			0.907 **	ns
FSC				ns

The temperature ranges from 21.9 to 25.0 °C and relative humidity ranges from 58.6 to 82.2% during the flowering period. Abbreviations; NVC, Nectar volume per catkin; NC, Nectar concentration; FSC, Free sugar content; SCPC, sugar content per catkin. * *p* < 0.05; ** *p* < 0.01; ns, non-significant, respectively.

**Table 3:** The composition of amino acid in nectar of *Castenea* spp. male flower.

Amino Acid (%)	CCK	CCJ	CDA	CMC	CSE	H Test *
Alanine	5.2 ± 1.1	3.8 ± 0.2	3.3 ± 0.5	6.2 ± 1.5	3.7 ± 1.4	
Arginine ^1^	0.6 ± 0.3	2.1 ± 0.6	1.3 ± 0.6	0.9 ± 0.2	1.1 ± 0.2	
Asparagine	15.5 ± 3.4 ^b^	12.9 ± 3.2 ^b^	15.1 ± 6.5 ^b^	32.4 ± 3.5 ^ab^	41.0 ± 0.6 ^a^	*p* = 0.048
Aspartic acid	8.9 ± 0.9	9.4 ± 2.4	6.0 ± 0.1	6.8 ± 0.9	6.4 ± 2.8	
GABA ^2^	3.8 ± 0.6 ^ab^	6.0 ± 0.8 ^a^	4.6 ± 0.9 ^ab^	3.5 ± 0.7 ^ab^	2.2 ± 0.7 ^b^	*p* = 0.047
Glutamic acid	13.2 ± 2.6	13.9 ± 3.6	10.9 ± 0.8	11.0 ± 1.8	7.8 ± 0.9	
Glutamine	4.2 ± 0.4 ^b^	6.0 ± 0.9 ^ab^	13.2 ± 2.0 ^a^	4.6 ± 0.5 ^b^	4.5 ± 0.7 ^ab^	*p* = 0.037
Glycine	1.0 ± 0.3	1.0 ± 0.3	1.1 ± 0.4	1.1 ± 0.2	0.8 ± 0.1	
Histidine ^1^	1.0 ± 0.1	1.4 ± 0.3	1.3 ± 0.2	0.9 ± 0.2	0.9 ± 0.2	
Isoleucine ^1^	1.3 ± 0.2	1.4 ± 0.7	1.3 ± 0.3	1.1 ± 0.0	0.6 ± 0.3	
Leucine ^1^	1.1 ± 0.2	1.1 ± 0.4	1.3 ± 0.4	0.8 ± 0.1	0.5 ± 0.3	
Lysine ^1^	0.7 ± 0.0	0.9 ± 0.3	0.9 ± 0.0	0.4 ± 0.0	0.5 ± 0.0	
Methionine ^1^	-	0.2 ± 0.1	0.4 ± 0.1	0.2 ± 0.1	0.1 ± 0.0	
Phenylalanine ^1^	0.6 ± 0.2	1.1 ± 0.4	0.6 ± 0.2	1.2 ± 0.4	0.7 ± 0.5	
Proline	29.6 ± 4.9 ^a^	22.3 ± 8.4 ^abc^	26.8 ± 4.3 ^ab^	7.3 ± 1.2 ^c^	18.0 ± 8.4 ^bc^	*p* = 0.049
Serine	5.1 ± 1.1	3.8 ± 0.4	4.7 ± 1.3	7.3 ± 0.2	5.7 ± 0.2	
Threonine ^1^	1.2 ± 0.3	1.9 ± 0.3	1.3 ± 0.7	1.5 ± 0.0	1.2 ± 0.2	
Tryptophan ^1^	5.5 ± 1.4 ^ab^	7.9 ± 2.4 ^ab^	3.4 ± 0.8 ^b^	10.6 ± 1.9 ^a^	2.9 ± 3.0 ^ab^	*p* = 0.042
Tyrosine	0.4 ± 0.0	0.9 ± 0.6	0.7 ± 0.3	0.3 ± 0.0	0.3 ± 0.1	
Valine ^1^	2.1 ± 0.3	2.0 ± 0.6	2.4 ± 0.4	2.2 ± 0.3	1.5 ± 0.5	
Total content (mg/L)	53.9 ± 12.6	76.2 ± 44.7	81.5 ± 31.9	118.1 ± 27.6	60.1 ± 9.6	

Data represent the mean ± SD (*n* = 3). * Kruskal-Wallis H test. The different letters within a row indicate significant differences among *Castanea* spp. based on Kruskal-Wallis H test with Dunn’s multiple comparison tests (*p* < 0.05). ^1^ Essential amino acid for honey bee, ^2^ Gamma-aminobutyric acid, a non-protein amino acid. Abbreviations; CCK, *C. crenata* (Korean cultivar ‘Mipung’); CCJ, *C. crenata* (Japanese cultivar ‘Ungi’); CDA, *C. dentata* (American chestnut); CMC, *C. mollissima* (Chinese chestnut); CSE, *C. sativa* (European chestnut).

**Table 4:** Identified VOCs in floral nectar of *Castenea* spp. (%).

VOCs	LRI	CCK	CCJ	CDA	CMC	CSE	H Test *
Styrene	887	-	0.58 ± 0.10	-	2.43 ± 1.82	-	
Benzaldehyde	960	2.24 ± 1.09	7.23 ± 3.58	38.92 ± 16.12	8.36 ± 0.66	40.81 ± 2.09	
Benzyl alcohol	1036	11.38 ± 0.98 ^a^	7.33 ± 0.36 ^ab^	4.74 ± 1.74 ^abc^	1.03 ± 0.09 ^c^	2.88 ± 0.49 ^bc^	*p* = 0.016
Benzeneacetaldehyde	1044	-	-	-	21.09 ± 3.15	2.07 ± 0.19	
Phenylethyl Alcohol	1062	18.54 ± 1.21	11.37 ± 0.87	-	0.65 ± 0.15	5.16 ± 0.51	
α-Methylbenzenemethanol,	1063	14.03 ± 0.71 ^a^	11.61 ± 0.58 ^b^	5.39 ± 1.20 ^bc^	7.76 ± 0.45 ^bc^	2.89 ± 0.40 ^c^	*p* = 0.011
Ethyl benzoate	1172	1.11 ± 0.13	-	-	-	-	
Benzoic acid, ethyl ester	1172	-	-	1.63 ± 0.16	-	-	
(*E*)-Cinnamaldehyde	1273	1.65 ± 0.47	-	-	-	-	
2’-Aminoacetophenone	1304	0.84 ± 0.07	-	-	-	-	
Acetophenone	1304	39.72 ± 2.72 ^a^	54.40 ± 1.85 ^a^	37.77 ± 10.45 ^a^	50.57 ± 3.35 ^a^	7.17 ± 0.53 ^b^	
Eugenol	1361	-	-	1.76 ± 0.36	-	-	
Hexanal	796	-	-	-	1.56 ± 0.62	-	
(*Z*)-3-Hexen-1-ol	856	7.50 ± 1.60 ^ab^	7.86 ± 0.92 ^ab^	3.00 ± 0.45 ^bc^	1.99 ± 0.15 ^c^	14.71 ± 1.35 ^a^	*p* = 0.014
(*E*)-2-Hexen-1-ol	869	-	-	-	-	3.46 ± 0.16	
1-Hexanol	871	-	1.20 ± 0.18	-	1.30 ± 0.11	-	
Ethyl hexanoate	1001	-	-	-	-	7.00 ± 3.34	
2-Ethyl-1-hexanol	1031	1.87 ± 0.21 ^b^	1.81 ± 0.04 ^b^	4.82 ± 0.80 ^ab^	2.06 ± 0.25 ^ab^	5.70 ± 0.44 ^a^	*p* = 0.034
1-Octanol	1073	-	-	-	-	3.51 ± 0.18	
Nonanal	1104	0.67 ± 0.00	-	1.80 ± 0.40	2.09 ± 1.03	2.46 ± 0.51	
1-nonanol	1173	-	-	-	-	4.52 ± 1.06	
(*Z*)-Geraniol	1230		0.44 ± 0.02	-	-	-	

Data represent the mean ± SEM (*n* = 3). -, compound absent. * Kruskal-Wallis H test. The different letters within a row indicate significant differences among *Castanea* spp. based on Kruskal-Wallis H test with Dunn’s multiple comparison tests (*p* < 0.05). Abbreviations; CCK, *C. crenata* (Korean cultivar ‘Mipung’); CCJ, *C. crenata* (Japanese cultivar ‘Ungi’); CDA, *C. dentata* (American chestnut); CMC, *C. mollissima* (Chinese chestnut); CSE, *C. sativa* (European chestnut).
